# Genetic variants of SOX9 contribute to susceptibility of gliomas among Chinese population

**DOI:** 10.18632/oncotarget.11679

**Published:** 2016-08-29

**Authors:** Liang Wang, Gang Li, Nan Liu, Zhen Wang, Xiaoshan Xu, Jing Qi, Dongni Ren, Pengxing Zhang, Yongsheng Zhang, Yanyang Tu

**Affiliations:** ^1^ Department of Neurosurgery, Tangdu Hospital, Fourth Military Medical University, Xi'an 710038, China; ^2^ Department of Experimental Surgery, Tangdu Hospital, Fourth Military Medical University, Xi'an 710038, China; ^3^ Department of Administrative, Tangdu Hospital, Fourth Military Medical University, Xi'an 710038, China

**Keywords:** SOX9, gliomas, polymorphism, genetic susceptibility

## Abstract

Gliomas make up about 80% of all malignant brain tumors, and cause serious public health problem. Genetic factors and environmental factors jointly caused the development of gliomas, and understanding of the genetic basis is a key component of preventive oncology. However, most genetic factors underlying carcinogenesis of gliomas remain largely unclear. In current study, we systematically evaluated whether genetic variants of SOX9 gene, a transcription factor that plays a central role in the development and differentiation of tumors, contribute to susceptibility of gliomas among Chinese population using a two-stage, case–control study. Results showed that SOX9 rs1042667 was significant associated with increased gliomas risk after adjusted by age, gender, family history of cancer, smoking status and alcohol status (Allele C vs A: OR=1.25; 95% CI=1.11-1.40; P=1.2×10^−4^). Compared with the carriers of genotype AA, both those of genotype AC (OR=1.37; 95% CI=1.13-1.66) and CC (OR=1.53; 95% CI=1.22-1.91) had significantly increased gliomas risk. This should be the first genetic association study which aims to evaluated the association between genetic variants of SOX9 and susceptibility of gliomas. Additional functional and association studies with different ethnic groups included are needed to further confirm our results.

## INTRODUCTION

Brain and central nervous system (CNS) malignancies refer to series of rarely occurred tumors [1]. According to the data of National Office for Cancer Prevention and Control in China, the estimated numbers of new cases and deaths in China were 101600 and 61000, respectively [2]. Gliomas, which are thought to arise from glial cells, make up about 80% of all malignant brain tumors [3]. Many risk factors have been identified as potential contributors to gliomas risk [4]. For example, ionizing radiation exposure, allergies or atopic disease(s) history, and potential influence of occupational exposures have been identified to be associated with gliomas risk [4–6]. Understanding of the genetic basis is a key component of preventive oncology. Genome-wide association studies (GWAS) have successfully identified a large number of common single-nucleotide polymorphisms (SNPs) influencing gliomas risk, while these SNPs only explain a small proportion of the genetic risk [7]. Furthermore, due to the common methodological challenges among gliomas studies include small sample sizes, heterogeneity of tumor subtypes, and retrospective exposure assessment, limited genetic loci were identified for gliomas risk [8–11].

SRY (Sex Determining Region Y)-Box (SOX) genes, which encode transcription factors belonging to the HMG (High Mobility Group) superfamily, have been identified to be associated with a large number of tumour types in vivo [12–14]. Among them, SOX9, which acts as a transcription factor that plays a central role in the development and differentiation of multiple cell lineages, were suggested to be associated with risk and prognosis of gliomas [15–17]. Rani et al [18] found MiR-145 functed as a tumor-suppressive RNA by targeting Sox9 and adducin 3 in human glioma cells. Genetic association studies could provide solid evidence for the Oncogenic role of SOX9 gene in human malignant gliomas. To present, no studies have evaluated that whether genetic variants of SOX9 contribute to susceptibility of gliomas. Given the biological importance of SOX9 gene and its implication in gliomas, we performed the current two-stage, case–control study to investigated the association between tag SNPs in the SOX9 gene and gliomas susceptibility among Chinese population.

## RESULTS

### Demographic characteristics of the subjects

In current study, totally 400 gliomas patients and 400 healthy control were recruited in the discovery stage, while 800 gliomas patients and 800 healthy control were recruited in the validation stage. Table 1 shows the comparison of gliomas patients and controls by selective characteristics in both the discovery stage and the validation stage. No significant variation in age, gender, family history of cancer, and smoking status was found between gliomas patients and healthy controls in two stages (P > 0.05), while the gliomas patients are more likely to be drinkers in the validation stage (P<0.001).

**Table 1:** Comparison of gliomas patients and controls by selective characteristics

Variables	Discovery stage			Validation stage		
	Cases (n=400)	Controls (n=400)	P value	Cases (n=800)	Controls (n=800)	P value
Age (years)	48.2±3.8	48.5±3.3	0.233	45.3±4.2	45.1±4.7	0.370
Gender (male)	244 (61.0%)	248 (62.0%)	0.771	480 (60.0%)	468 (58.5%)	0.542
Family history of cancer	86 (21.5%)	75 (18.8%)	0.332	160 (20.0%)	131 (16.4%)	0.060
Smoking status						
Ever	100 (25.0%)	97 (24.2%)	0.806	162 (20.2%)	132 (16.5%)	0.053
Never	300 (75.0%)	303 (75.8%)		638 (79.8%)	668 (83.5%)	
Alcohol status						
Ever	114 (28.5%)	104 (26.0%)	0.435	238 (29.8%)	160 (20.0%)	**P<0.001**
Never	326 (81.5%)	336 (84.0%)		562 (70.2%)	640 (80.0%)	

### Associations between SOX9 gene polymorphisms and gliomas risk in the discovery stage

Totally seven tagSNPs (rs1042667, rs918080, rs16977091, rs9893662, rs7502198, rs6501522, and rs9915657) are selected using the SNPinfo web-based software. As shown in Table 2, the genotype frequencies of the selected SNPs and their associations with gliomas risk are presented. All of the genotype distributions for the seven tag SNPs were consistent with the HWE (P > 0.05). Our results indicated that SOX9 rs1042667 was significant associated with increased gliomas risk (Allele C vs A: OR=1.26; 95% CI=1.04–1.54; P=0.019). Compared with the carriers of genotype AA, both those of genotype AC (OR=1.45; 95% CI=1.03–2.04) and CC (OR=1.55; 95% CI=1.06–2.28) had significantly increased gliomas risk. However, no significant trend was detected for rs918080, rs16977091, rs9893662, rs7502198, rs6501522, and rs9915657.

**Table 2:** Association between SOX9 gene polymorphisms and the risk of gliomas in the discovery stage

SNPs	Subject	Genotype (N)			OR (95 % CI)^1^			P value
		11	12	22	2 vs 1	12 vs 11	22 vs 11	
rs1042667	Case	91	195	114	1.26 (1.04–1.54)	1.45 (1.03–2.04)	1.55 (1.06–2.28)	**0.019**
	Control	122	180	98				
rs918080	Case	271	100	29	1.20 (0.93-1.55)	0.99 (0.72-1.37)	1.87 (1.00-3.50)	0.156
	Control	280	104	16				
rs16977091	Case	310	72	18	0.92 (0.69-1.22)	0.75 (0.53-1.06)	1.43 (0.68-3.01)	0.564
	Control	296	92	12				
rs9893662	Case	193	140	67	1.20 (0.97-1.48)	0.91 (0.68-1.24)	1.65 (1.07-2.54)	0.087
	Control	200	158	42				
rs7502198	Case	122	210	68	1.20 (0.88-1.64)	1.30 (0.85-1.98)	1.14 (0.94-1.39)	0.187
	Control	140	200	60				
rs6501522	Case	250	116	34	1.07 (0.84-1.36)	0.92 (0.68-1.25)	1.41 (0.82-2.45)	0.549
	Control	250	126	24				
rs9915657	Case	252	108	40	1.08 (0.85-1.36)	0.84 (0.61-1.14)	1.57 (0.93-2.66)	0.512
	Control	248	127	25				

1 adjusted for Age, gender, family history of cancer, smoking status and alcohol status

### Validation analysis of the association between SOX9 rs1042667 and gliomas risk

To validate the positive findings, the association between SOX9 rs1042667 and gliomas risk was evaluated in an independent stage (Table 3). The genotype distribution for rs1042667 was also consistent with the HWE in the stage (P > 0.05). The results showed that SOX9 rs1042667 was also significant associated with increased gliomas risk (Allele C vs A: OR=1.24; 95% CI=1.08-1.42; P=0.002). when merged two stages together, SOX9 rs1042667 was significant associated with increased gliomas risk (Allele C vs A: OR=1.25; 95% CI=1.11-1.40; P=1.2×10^−4^). Compared with the carriers of genotype AA, both those of genotype AC (OR=1.37; 95% CI=1.13-1.66) and CC (OR=1.53; 95% CI=1.22-1.91) had significantly increased gliomas risk. We also conducted stratified analysis by alcohol status. However, the results didn't changed materially (Table 4).

**Table 3:** Genotype frequencies of rs1042667 and association with risk of gliomas in validation stage and the merged results

rs1042667	Cases (n=800)	Controls (n=800)	OR^1^ (95% CIs)	*P* value
**validation stage**				
AA	185 (23.1%)	236 (29.5%)	Reference	
AC	390 (48.8%)	374 (46.8%)	1.33 (1.05-1.69)	**0.019**
CC	225 (28.1%)	190 (23.7%)	1.51 (1.15-1.98)	**0.003**
Additive model			1.24 (1.08-1.42)	**0.002**
**Merged results**				
AA	276 (23.0%)	358 (29.8%)	Reference	
AC	585 (48.7%)	554 (46.2%)	1.37 (1.13-1.66)	**1.6×10^−3^**
CC	339 (28.3%)	288 (24.0%)	1.53 (1.22-1.91)	**1.8×10^−4^**
Additive model			1.25 (1.11-1.40)	**1.2×10^−4^**

1 adjusted for Age, gender, family history of cancer, smoking status and alcohol status

**Table 4:** Association of rs1042667 with risk of gliomas stratified by Alcohol status

rs1042667	Cases (n=1200)	Controls (n=1200)	OR^1^ (95% CIs)	*P* value
**Ever drinkers**	352	264		
AA	77 (21.9%)	80 (30.3%)	Reference	
AC	173 (49.1%)	121 (45.8%)	1.48 (1.05-1.64)	**0.046**
CC	102 (29.0%)	63 (23.9%)	1.68 (1.08-2.62)	**0.021**
Additive model			1.31 (1.05-1.64)	**0.018**
**Never drinkers**	848	936		
AA	199 (23.5%)	278 (29.7%)	Reference	
AC	412 (48.6%)	433 (46.3%)	1.33 (1.06-1.67)	**0.014**
CC	237 (27.9%)	225 (24.0%)	1.47 (1.14-1.90)	**3.2×10^−3^**
Additive model			1.23 (1.07-1.40)	**2.5×10^−3^**

1 adjusted for Age, gender, family history of cancer, and smoking status

## DISCUSSION

In current study, we systematically evaluated whether genetic variants of SOX9 contribute to susceptibility of gliomas among Chinese population using a two-stage, case–control study. Results strongly indicated that SOX9 rs1042667 was significant associated with 1.25-flod increased gliomas risk, after adjusted by age, gender, family history of cancer, smoking status and alcohol status. To be best of our knowledge, this should be the first genetic association study which aims to evaluated the association between genetic variants of SOX9 and susceptibility of gliomas.

The prognosis for patients with gliomas is often very poor, only ~2% of which aged 65 years or older [19]. Previous studies revealed that genetic, behavioral, environmental and developmental contributed to gliomas risk, although only exposure to therapeutic or high-dose radiation was firmly established [20]. Studies of genetic syndromes, familial aggregation, linkage and mutagen sensitivity, which identified specific candidate genes including APC, hMLH1, hMSH2, PMS2, PTEN, NF1, NF2, et al, indicated the genetic susceptibility to gliomas [20–22]. Furthermore, genome-wide association studies (GWASs) show that common genetic variation contributes to the heritable risk of gliomas, and identify some new gliomas susceptibility loci, however, these only account for a small proportion of gliomas cases [8]. Additional studies, which aims to find more potential susceptibility loci to reveal the underlying genetic basis for gliomas, are needed and may yield increased insight into the development of this malignancy.

SOX9 gene is located on a gene desert on 17q24 in humans, and could recognize the CCTTGAG sequence along with other members of the HMG-box class DNA-binding proteins [23, 24]. Sox9 is required for the early differentiation of the prostate bud epithelia, and fully involved in the carcinogenesis, differentiation, and invasion prostate cancer through reactivating the WNT/beta-catenin signaling that mediates ductal morphogenesis [25–28]. Besides, Sox9 was also associated the carcinogenesis, development and prognosis of colorectal cancer, lung cancer, melanoma, hepatocellular carcinoma, skin tumors, and cervical cancer [29–37]. Pop et al [38] found a homozygous nonsense mutation in SOX9 in the dominant disorder campomelic dysplasia. Zhang et al [39] identified that multiple genetic variants mapping to a unique enhancer looping to the SOX9 oncogene could account for the risk associated with 17q24.3 locus of prostate cancer.

In current study, we confirmed that SOX9 rs1042667 was significantly associated with increased gliomas risk (Allele C vs A: OR=1.25; 95% CI=1.11-1.40; P=1.2×10^−4^). Using Quanto software (http://biostats.usc.edu/Quanto.html), we found we have 97% statistical power to get such an association. Rs1042667 located at the 3′ UTR region of the SOX9 gene. Using RegulomeDB [40, 41], rs1042667 was identified to be likely to affect binding capacity with other genes and transcription factors. While it was reported to be associated with the expression of AC005152.2 in Heart Left Ventricle, and could alter regulatory motifs, including GR_disc4, TATA_disc1, THAP1_disc2, and YY1_disc1, when analyzed by HaploReg v4.1 [42]. It was also predicted to be the binding site of transcription factors (ATF6_01, CACD_01, CDPCR3_01, et al) using the SNPinfo package (https://snpinfo.niehs.nih.gov/cgi-bin/snpinfo/snpfunc.cgi). Overall, our findings provided evidence for the important role of SOX9 gene in the tumorigenesis of gliomas.

Several limitations should be considered when interpreting the results of this study. First, due to the natural of case-control study design, selection and information bias might be unavoidable. We have tried our best to eliminate this by matching the control by age, gender, and race from the same hospital, a face to face interview, and unified training of the investigators. Second, gene-environment interactions was not detected in current study, which may be caused by the relatively not very large sample size.

Conclusively, the present two-stage, case-control study revealed that SOX9 rs1042667 was associated with increased gliomas risk in a Chinese population. Further functional and genetic association studies in larger population, with different ethnic groups included, are warranted to further validate our results and explore the possible mechanism of SOX9 gene in the carcinogenesis and development of gliomas.

## MATERIALS AND METHODS

### Subjects

All of the gliomas cases received treatments from the Tangdu Hospital until November 2015, we recruited 1,200 genetically unrelated Chinese patients with newly diagnosed and histopathologically confirmed primary gliomas. We also included a total of 1,200 age-, gender-, race-matched, cancer-free volunteers recruited from the same hospital. The response rate was approximately 95% and 92% for gliomas subjects and cancer-free controls, respectively. All the participants have no previous history of cancer and CNS-related diseases. Structured-interviewer-administered questionnaires were used to collect data on demographic characteristics and potential gliomas risk factors. The study was approved by the Institutional Review Board of Tangdu Hospital. All of the participants provided written informed consent by themselves or their guardians.

### SNP selection and genotyping

**Figure 1:**
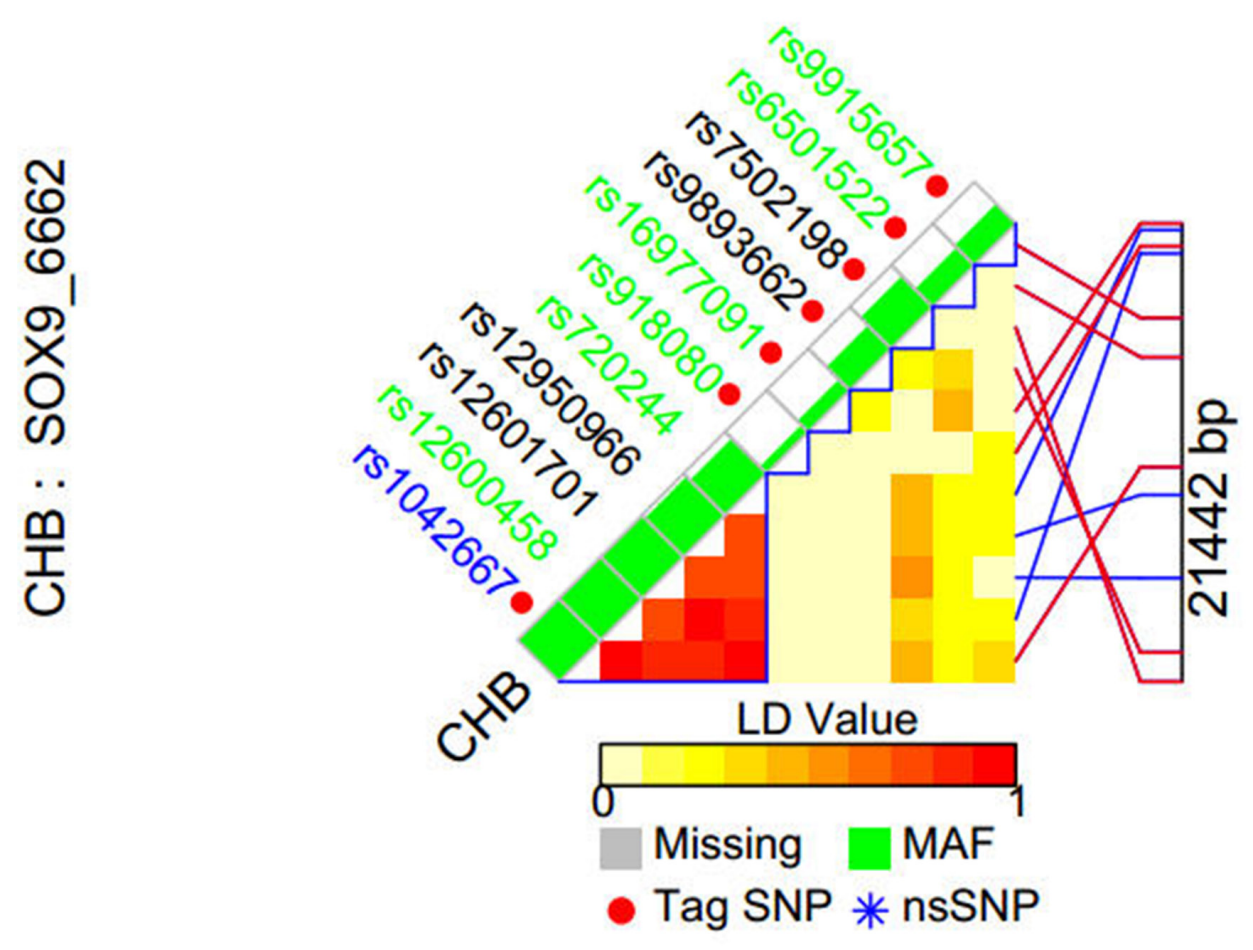
tag SNP selection of the SOX9 gene

The tag SNPs of SOX9 gene and its 10kb flanking region were selected using SNPinfo (http://snpinfo.niehs.nih.gov/) based on the criteria of minor allele frequency(MAF) >5% for Chinese Han subjects; Seven tag SNPs in the SOX9 gene that met the criteria were chosen in this study (Figure 1). Genomic DNAs were extracted by Qiagen DNA blood kit (Qiagen, Hilden, Germany) from whole blood samples collected from all subjects. The extraction of genomic DNAs was performed following the manufacturer's protocols. SNP genotyping was performed by Sequenom MassArray iPLEX platform (Sequenom Inc., San Diego, CA, USA). To validate the accuracy of genotyping results and for quality control, approximately 10% of the samples were randomly selected and genotyped with sequencing. Results showed that the concordance for the quality control samples was 100%.

### Statistical analysis

All the data was analyzed with SPSS software version 13.0 (SPSS Inc, Chicago, IL, USA). All statistical tests were two-sided, with a significance level of P < 0.05. The chi-square test was used to compare the difference in gender, family history of cancer, smoking status and alcohol status between gliomas patients and healthy controls, while Student's paired t test was performed to compare the difference in age between gliomas patients and healthy controls. Genotypic frequencies in controls for each SNP were tested for departure from HWE using goodness-of-fit χ2 test. Odds ratios and corresponding 95% confidence intervals (CIs) were used to estimate the association between selected polymorphisms and gliomas risk. Adjusted ORs were calculated by multivariate analysis with unconditional logistic regression, with adjustment for age, gender, family history of cancer, smoking status and alcohol status.
